# West Nile Virus Antibody Prevalence in Horses in Ukraine

**DOI:** 10.3390/v5102469

**Published:** 2013-10-04

**Authors:** Ute Ziegler, Artem Skrypnyk, Markus Keller, Christoph Staubach, Maksym Bezymennyi, Armando M. Damiani, Nikolaus Osterrieder, Martin H. Groschup

**Affiliations:** 1 Friedrich-Loeffler-Institut, Federal Research Institute for Animal Health, Institute of Novel and Emerging Infectious Diseases, Südufer 10, D-17493 Greifswald-Insel Riems, Germany; E-Mails: ute.ziegler@fli.bund.de (U.Z.); markus.keller@fli.bund.de (M.K.); martin.groschup@fli.bund.de (M.H.G.); 2 Institute of Veterinary Medicine, National Academy of Agrarian Sciences of Ukraine, Donetska Str. 30, Kyiv, 03151 Ukraine; E-Mails: artemskrypnyk@yahoo.com (A.S.); nomax@ukr.net (M.B.); 3 Friedrich-Loeffler-Institut, Federal Research Institute for Animal Health, Institute of Epidemiology, Seestraße 55, D-16868 Wusterhausen, Germany; E-Mail: christoph.staubach@fli.bund.de (C.S.); 4 Institut für Virologie, Zentrum für Infektionsmedizin, Freie Universität Berlin, Philippstr. 13, D-14163 Berlin, Germany; E-Mails: adamiani@zedat.fu-berlin.de (A.M.D.); no.34@fu-berlin.de (N.O.)

**Keywords:** West Nile virus, neutralization, ELISA, serology, horse, Ukraine, prevalence, flavivirus

## Abstract

West Nile virus (WNV) is a mosquito-borne virus of global importance. Over the last two decades, it has been responsible for significant numbers of cases of illness in humans and animals in many parts of the world. In Ukraine, WNV infections in humans and birds were first reported more than 25 years ago, yet the current epidemiological status is quite unclear. In this study, serum samples from over 300 equines were collected and screened in order to detect current WNV activity in Ukraine with the goal to estimate the risk of infection for humans and horses. Sera were tested by enzyme-linked immunosorbent assay (ELISA) and virus neutralization assay (NT) to detect WNV-specific antibodies. The results clearly revealed that WNV circulates in most of the regions from which samples were obtained, shown by a WNV seroprevalence rate of 13.5% of examined horses. This is the first topical report indicating the presence of WNV infections in horses in Ukraine, and the results of this study provide evidence of a widespread WNV circulation in this country.

## 1. Introduction

West Nile virus (WNV) is an arbovirus that is maintained in an enzootic cycle between ornithophilic mosquitoes and certain wild bird species. WNV is the most widespread flavivirus known, was first isolated in Uganda in 1937, and has been found on all continents [1,2], except for Antarctica. Distinct wild bird species serve as major reservoir hosts, although even under natural conditions a variety of mammalian and reptilian species are susceptible [3]. Likewise, a variety of different bird and mosquito species are involved in the WNV life cycle in various regions of the world [4]. WNV infections in humans and equines can lead to febrile illness, which in some cases will progress to encephalitis, occasionally with fatal outcome [5]. Outside of the African continent, WNV usually causes clinical cases only sporadically in humans and equines in Mediterranean countries, the Middle East, Romania and Russia [1,6,7,8]. WNV encephalitis cases were first reported in humans and horses in the Camargue region of France in the early 1960s [9] and WNV has since been observed repeatedly in the central part of Europe [10]. Until the mid1990s, WNV was considered a minor risk for humans and horses as —with some regional exceptions —it occurred only sporadically [11]. The first major WNV epidemic in Eastern Europe was observed in Romania in 14 districts of the lower Danube valley and in Bucharest with more than 400 clinical cases in 1996 [6]. In the late 1990s, outbreaks of WNV fever with high numbers of infected humans were also observed in Israel and southern Russia [12]. WNV infections became a major concern for veterinary public health after its introduction into the New World and rapidly spread to almost all countries on the American continent over the following years [13]. Large WNV outbreaks affecting humans and equines have been reported most recently in Greece [14], and WNV lineage 2 sequences could be demonstrated in human blood donors, *Culex* mosquitoes, wild birds and sentinel chickens. In the summer of 2012, evidence of WNV circulation was also found in Slovakia [15] and in various Balkan countries, including Serbia, Kosovo, Macedonia and Croatia [16]. Major outbreaks in humans and horses have also been observed over the past few years in the Mediterranean area, e.g. in Italy; however, preferentially WNV lineage 1 sequences have been detected [17]. Recently, WNV lineage 2 viruses have also been detected in wild birds in Sardinia [18] and in northeastern Italy. 

In Ukraine, the earliest reports confirming the presence of WNV infections in humans and birds date back to the 1970s. In 1974, Sidenko *et al*. [19] described human WNV infections and the accompanying neurological signs, as well as WNV specific antibodies in wild birds and farm animals in the southwestern USSR. In 1985, 38 human cases, 16 of them with neurological manifestations, were recorded in the Transcarpathian region (Ukrainian SSR). The course of disease was benign [20]. At the same time (1980s), a WNV strain was detected in the blood and internal organs of a wild bird (Rook, *Corvus frugileus*) in the territory of the Black Sea [21]. A recently conducted study on arbovirus infections showed new active natural foci in the forest-steppe zone of Ukraine and a close etiological relation was found between acute seasonal febrile diseases and the above-mentioned arbovirus. Besides tick-borne encephalitis, WNV disease, therefore, is the leading arboviral infection in the forest-steppe zone of Ukraine [22]. 

Recently, clinical WNV infections have been detected in patients in Ukraine in the summer of 2011 (eight WNV cases in three different regions/oblasts) and in 2012 (12 human cases in one region (Poltavska)) [23,24,25]. However, little is known about the extent of WNV infections in horses in Ukraine at present. The aim of the monitoring study (two-year-analysis) presented here was to investigate the prevalence of WNV specific antibodies in horse sera from 14 different Ukrainian regions (oblasts) by means of enzyme-linked immunosorbent assay (ELISA) and virus neutralization assays (NT). The results provide relevant information on the current WNV situation in Ukraine, involving an infection risk for humans and horses.

## 2. Results and Discussion

**Figure 1:**
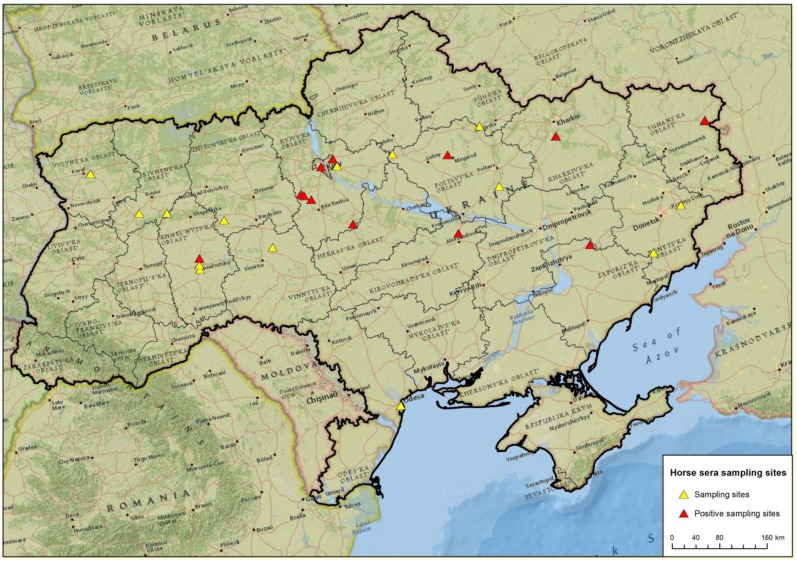
Sampling sites of the horse sera with focus on known West Nile virus (WNV) risk areas.

Horse sera from 310 animals kept in 14 different Ukrainian regions were collected in 2010 and 2011 (Figure 1, Table 1). Sera were screened by a commercial WNV competition ELISA, which essentially detects flavivirus antibodies. Therefore ELISA-positive results were verified by virus neutralization tests (NT) to identify WNV or cross-reactivity specific reactions. 

**Table 1:** Distribution of serum samples by regions (oblasts).

**Ukraine**	**Region**	Investigated sera	by WNV-ELISA	only WNV-NT
**1**	Donetska	11	11	-
**2**	Kharkivska	21	21	-
**3**	Khmelnytska	39	38	1
**4**	Kyivska	118	113	5
**5**	Kirovohradska	59	52	7
**6**	Luhanska	20	20	-
**7**	Odeska	1	1	-
**8**	Poltavska	6	6	-
**9**	Rivnenska	13	11	2
**10**	Sumska	2	2	-
**11**	Vinnytska	2	2	-
**12**	Volynska	10	10	-
**13**	Zaporizka	6	6	-
**14**	Zhytomyrska	2	2	-
**Total**		**310**	**295**	**15**

### 2.1. WNV competition Enzyme-linked immunosorbent assay (ELISA).

**Figure 2:**
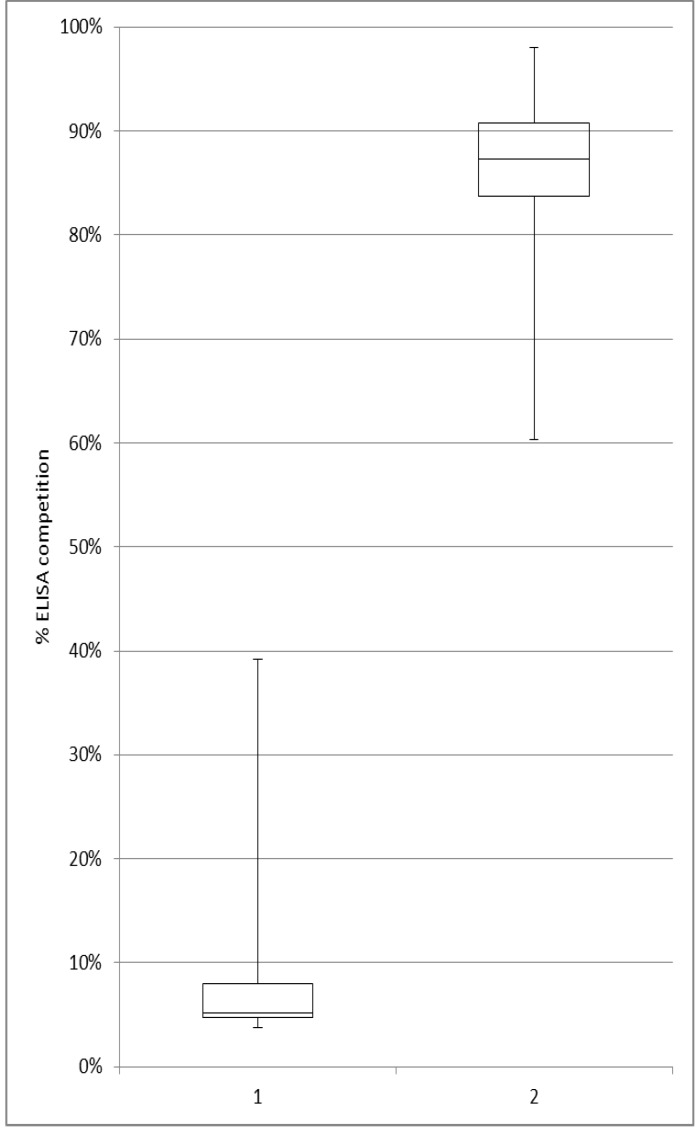
Competitive ELISA (S/N%) results of all 295 investigated horse sera. (1 = reactive samples, 2 = negative samples)

From the 310 sera, 295 samples were investigated using the competitive WNV ELISA. Volumes of the remaining 15 samples were so low (<50 µl) that they were only used for different NT (Table 1). The ELISA results showed a high rate of seropositive horses in different regions of Ukraine (Table 2). A total of 49 out of the 295 sera were reactive (Figure 2). Resulting in an overall ELISA prevalence rate of 16.6%., WNV antibody-positive animals were discovered in seven of the 14 investigated Ukrainian regions and high ELISA prevalence rates between 20% and 25% were determined for the Luhanska, Kharkivska and Kirovohradska regions. Even higher prevalence rates were obtained for Poltavska (33%) and Zaporizka (83%), with the caveat, however, that available sample numbers were small (n=6) (Table 2). All 49 ELISA-positive samples were investigated further by the different NT to exactly determine the virus resulting in ELISA seropositivity.

**Table 2:** Competitive ELISA results stratified by region and regional antibody prevalence.

**Ukraine**	**Region**	by ELISA	comp. ELISA negative	comp. ELISA reactive (doubtful or positive)	ELISA prevalence
**1**	Donetska	11	11	-	-
**2**	Kharkivska	21	16	5	23.80 %
**3**	Khmelnytska	38	37	1	2.63 %
**4**	Kyivska	113	94	19	16.81 %
**5**	Kirovohradska	52	39	13	25.00 %
**6**	Luhanska	20	16	4	20.00 %
**7**	Odeska	1	1	-	-
**8**	Poltavska	6	4	2	33.33 %
**9**	Rivnenska	11	11	-	-
**10**	Sumska	2	2	-	-
**11**	Vinnytska	2	2	-	-
**12**	Volynska	10	10	-	-
**13**	Zaporizka	6	1	5	83.33 %
**14**	Zhytomyrska	2	2	-	-
**Total**		**295**	**246**	**49**	**16.61 %**

### 2.2. Virus neutralization assays (NT)

ELISA results were generally confirmed by NT results. In total, 68 horse sera (49 initially ELISA positive sera plus the 15 sera of which only small volumes were available and four additional sera, randomly selected) were investigated in a WNV neutralization assay. The ELISA results were confirmed for 42 out of the 49 ELISA-positive sera by WNV-NT, with neutralization titers ranging between 1:10 and 1:640 (Table 3). From the 15 sera with small volumes for which no ELISA results were available, three sera contained WNV-specific antibodies. One sample was excluded due to serum toxicity for cells. 

Another seven sera with positive ELISA and negative WNV-NT results, as well as sera with weak positive WNV-NT results, were eventually run in a TBEV neutralization test (NT) to check for cross-reactivity. False positive ELISA results caused by TBE cross-reactive antibodies had been observed in earlier serological studies looking at horses from Germany and Austria [26,27,28] which impede the interpretation of the ELISA results. However, no TBEV-NT positive horse serum was found among the Ukrainian horse sera. Although Lozyns'kyĭ and Vynohrad (1998 [22]) described the occurrence and wide distribution of tick-borne encephalitis in the forest-steppe zone of Ukraine, our sample panel revealed no incidence of a TBEV infection.

### 2.3. Indirect WNV IgM ELISA

ELISA-positive sera with clear NT negative or weak positive NT results, as well as sera, of which only small sample volumes (< 50µl) were available, were also run in a commercially available IgM ELISA to detect recent WNV infections. This capture ELISA detects WNV specific IgM antibodies in horse sera for up to 3 months post infection. However, none of the 23 investigated samples contained WNV IgM antibodies (data not shown). Therefore there was no evidence for recent WNV infections in these samples.

**Table 3:** ELISA and neutralization assay results of 68 horse samples. (* not enough serum material for comp. ELISA, < 50µl)

Laboratory sample no.	comp. WNV-ELISA S/N%	WNV-ELISA results	NT-ND_50 (WNV lineage 2)_	WNV-NT results	NT-ND_50 (TBEV-Neudoerfl)_	TBEV-NT results
**ELISA and WNV-NT positive** (n=42)						
33	14.19%	positive	10	weak positive	< 10	negative
44	12.28%	positive	15	weak positive	< 10	negative
53	6.18%	positive	60	positive	< 10	negative
54	4.98%	positive	480	positive	< 10	negative
58	6.35%	positive	640	positive	< 10	negative
59	5.09%	positive	320	positive	< 10	negative
64	4.73%	positive	640	positive	< 10	negative
68	4.94%	positive	160	positive	< 10	negative
69	4.87%	positive	640	positive	< 10	negative
71	4.98%	positive	240	positive	< 10	negative
72	4.83%	positive	160	positive	< 10	negative
75	5.90%	positive	30	positive	< 10	negative
88	5.53%	positive	40	positive	< 10	negative
96	5.97%	positive	40	positive	< 10	negative
97	4.91%	positive	160	positive	< 10	negative
105	5.77%	positive	60	positive	< 10	negative
107	4.73%	positive	160	positive	< 10	negative
108	4.67%	positive	160	positive	< 10	negative
123	7.86%	positive	80	positive	< 10	negative
124	4.81%	positive	80	positive	< 10	negative
128	4.15%	positive	160	positive	< 10	negative
131	3.88%	positive	80	positive	< 10	negative
146	8.79%	positive	15	weak positive	< 10	negative
149	4.31%	positive	20	positive	< 10	negative
158	4.04%	positive	80	positive	< 10	negative
160	3.78%	positive	320	positive	< 10	negative
166	4.55%	positive	120	positive	< 10	negative
187	4.63%	positive	180	positive	< 10	negative
208	6.09%	positive	10	weak positive	< 10	negative
217	7.84%	positive	30	positive	< 10	negative
228	4.44%	positive	120	positive	< 10	negative
232	10.92%	positive	10	weak positive	< 10	negative
288	4.48%	positive	320	positive	< 10	negative
289	9.16%	positive	60	positive	< 10	negative
292	5.09%	positive	40	positive	< 10	negative
297	5.35%	positive	480	positive	< 10	negative
312	5.12%	positive	60	positive	< 10	negative
313	4.92%	positive	160	positive	< 10	negative
330	7.98%	positive	80	positive	< 10	negative
336	4.18%	positive	120	positive	< 10	negative
337	4.44%	positive	160	positive	< 10	negative
343	7.08%	positive	10	weak positive	< 10	negative
**ELISA positive and WNV-NT negative** (n=7)						
98	34.70%	positive	< 10	negative	< 10	negative
106	26.64%	positive	< 10	negative	< 10	negative
183	14.07%	positive	< 10	negative	< 10	negative
230	23.58%	positive	< 10	negative	< 10	negative
250	30.98%	positive	< 10	negative	< 10	negative
295	25.88%	positive	< 10	negative	< 10	negative
328	39.16%	positive	< 10	negative	< 10	negative
**ELISA negative and WNV-NT positive** (n=4)						
209	91.31%	negative	30	positive	< 10	negative
210	86.89%	negative	120	positive	< 10	negative
212	90.21%	negative	10	weak positive	< 10	negative
214	93.03%	negative	320	positive	< 10	negative
**Low volume ➔ no ELISA, WNV-NT positive** (n=3)						
126	n.d.	*	80	positive	< 10	negative
127	n.d.	*	60	positive	< 10	negative
162	n.d.	*	60	positive	< 10	negative
**Low volume ➔ no ELISA, WNV-NT negative** (n=11+1 not evaluable)						
27	n.d.	*	< 10	negative	< 10	negative
76	n.d.	*	< 10	negative	< 10	negative
100	n.d.	*	not evaluable, because of serum toxicity for cells			
125	n.d.	*	< 10	negative	< 10	negative
129	n.d.	*	< 10	negative	< 10	negative
130	n.d.	*	< 10	negative	< 10	negative
138	n.d.	*	< 10	negative	< 10	negative
139	n.d.	*	< 10	negative	< 10	negative
163	n.d.	*	< 10	negative	< 10	negative
179	n.d.	*	< 10	negative	< 10	negative
180	n.d.	*	< 10	negative	< 10	negative
186	n.d.	*	< 10	negative	< 10	negative

**Figure 3:**
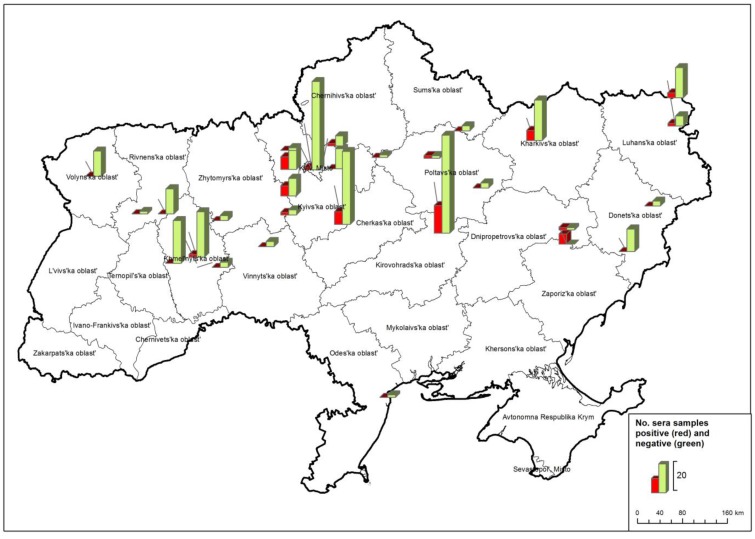
Number of WNV positive sera (red columns = both in ELISA and neutralization test (NT)) in horses from different Ukrainian regions. ELISA negative samples are depicted in green columns.

In summary, our results show that WNV circulates in large parts of Ukraine. In total, 42 horse sera (Table 3) tested positive for WNV antibodies as determined by both ELISA and NT. These samples were collected in seven regions, namely Kharkivska, Khmelnytska, Kyivska, Kirovohradska, Luhanska, Poltavska and Zaporizka (Figure 3, Table 4).

**Table 4:** Distribution of the ELISA and NT positive samples.

Ukraine	region	comp. ELISA positive and WNV-NT positive	WNV-NT ND_50_ titres
**1**	Donetska	-	-
**2**	Kharkivska	4	10 - 160
**3**	Khmelnytska	1	10
**4**	Kyivska	17	10 - 640
**5**	Kirovohradska	11	30 - 160
**6**	Luhanska	3	15 - 120
**7**	Odeska	-	-
**8**	Poltavska	1	40
**9**	Rivnenska	-	-
**10**	Sumska	-	-
**11**	Vinnytska	-	-
**12**	Volynska	-	-
**13**	Zaporizka	5	60 - 480
**14**	Zhytomyrska	-	-
		**42**	

The overall findings reported here are consistent with those from previous WNV seroprevalence studies in horses, mosquitoes and wild birds [7,19,21,29,30] and humans [19,20,29] in Ukraine. Most recently, high infection rates have also been found in humans in Romania and the Russian Federation, which are immediate neighbors of Ukraine [6,12]. Large parts of the Ukrainian landscape (rivers, wetlands and lowlands) and the country`s climatic conditions (wet winters and springs followed by excessive heat in the summers) are commensurate with environmental conditions necessary for productive circulation of WNV in wildlife. Moreover, Ukraine is located on major migratory bird flyways connecting Africa and The Middle East with Eurasia [31]. WNV endemics establish themselves particularly in urban regions with open water drainage systems and improper basic sanitation [32]. During the time of sampling, no clinical WNV cases were reported in horses, whereas clinical cases were confirmed in humans in 2011 and 2012 in Ukraine [23,24,25]. 

To exclude serological cross-reactions leading to false WNV seropositivity, differential diagnostic assays were conducted. As the WNV ELISA used also detects cross-reacting antibodies induced by other flaviviruses, e.g. TBEV [26,27,28], all sera negative in the WNV NT were also screened using a TBEV NT as well as a Japanese encephalitis virus (JEV) NT, all with negative outcomes (data not shown). The reason for ELISA reactivity of the seven sera (positive WNV ELISA, but negative in all conducted NTs, Table 3) is unclear at present. Besides measuring different properties of antibodies in the assay systems, this may indicate circulation of an as-yet-unknown flavivirus in Ukraine. 

## 3. Experimental Materials and Methods

*Serum samples.* Blood samples were collected from 310 randomly selected healthy horses, which were kept in 14 regions of Ukraine during 2010 and 2011 (Table 1). Horses had no known clinical history of a previous WNV infection. Sera were kept at –20 ºC until use. 

*ELISA.* Sera were screened for WNV specific antibodies using a commercially available competition ELISA, which allows the species-independent recognition of WNV antibodies against the PrM- and E envelope protein (ID Screen^®^ West Nile Competition, IDVet, Montpellier, France). The ELISA cut-off is defined by the residual binding ratios (S/N%-value); sera with S/N ratios of 40% and lower are positive, while samples with S/N ratios of more than 50 are considered WNV antibody-negative. S/N values of 40–50% are inconclusive. Additionally, a commercially available IgM capture ELISA was used (IDEXX IgM WNV Ab Test, IDEXX Europe B.V., Hoofddorp, the Netherlands) to detect recent WNV infection in horses.

*NT.* ELISA results were confirmed by virus neutralization test carried out under biosafety level 3 conditions and using Vero cells on 96-well plates as described earlier [33]. Test serum dilutions (20 µl starting heat-inactivated serum material) were pre-incubated with 100 TCID_50_ of WNV strain Austria (lineage 2, Accession no. HM015884, kindly provided by Dr. N. Nowotny, Institute of Virology, University of Veterinary Medicine, Vienna). All samples were run in duplicate and NT titers were calculated after inspection of the assay at 6 to 7 days after infection, depending on the cytopathic effects in the infected control wells. The neutralizing antibody titer was defined as the neutralization dose 50% (ND_50_), i.e. the maximum dilution, which inhibited cytopathic effects in 50% of the wells according to the Behrens-Kaerber method. ND_50_ values of above 10 were considered positive. The TBEV serum neutralization test was carried out following the same protocol, except that the TBEV strain Neudoerfl (kindly provided by Dr. F. Hufert, Institute for Virology, Göttingen Germany; GenBank accession no. U27495) was used. Furthermore, JEV-NT was carried out using the same procedure and using JEV strain Nakayama (GenBank accession no. EF571853).

*Maps.* GIS-Analysis of the sampling sites and of the results was performed by using the ArcGIS Arview 10.0 software (ESRI, Redlands, CA, USA) and displayed using a Lambert conformal conic coordinate system.

## 4. Conclusions

WNV specific antibodies were detected in sera from horses originating from seven Ukrainian regions, thus representing a substantial part of the country’s territory (Figure 1), and the mean sero-prevalence rate for WNV was 13.5%. However, IgM ELISA analyses on selective sera did not reveal any evidence of recent infections. Samples were unsuitable for the demonstration of WNV genomes.

This is the first up-to-date report indicating WNV infections prevalence in horses in Ukraine, and these results provide evidence of widespread WNV circulation in this country. The results will help to determine the risk of infection for humans and to control WNV transmission. Surveillance studies in humans, vectors and animals are needed to better define endemic areas. 
